# Acute Exposure to Key Aquaculture Environmental Stressors Impaired the Aerobic Metabolism of *Carassius auratus gibelio*

**DOI:** 10.3390/biology9020027

**Published:** 2020-02-10

**Authors:** Zongli Yao, Xiaoying Zhang, Qifang Lai, Kai Zhou, Pengcheng Gao

**Affiliations:** Engineering Research Center for Saline-alkaline Water Fisheries, Sino-US Joint Laboratory of Aquatic Animal Physiology, Key Laboratory of East China Sea Fishery Resources Exploitation, Ministry of Agriculture and Rural Affairs, East China Sea Fisheries Research Institute, Chinese Academy of Fishery Sciences, Shanghai 200090, China; yaozongli@hotmail.com (Z.Y.); zhangxy8123@163.com (X.Z.); zhoukai69@126.com (K.Z.); legend04fsefan@163.com (P.G.)

**Keywords:** aerobic metabolism, ammonia, nitrite, pH, hypoxia, *Carassius auratus gibelio*

## Abstract

*Carassius auratus gibelio* is an omnivore favored for its flavor and is commonly used as a benthic species in traditional pond polyculture. This study investigated the effects of common aquaculture stressors, such as high ammonia, high nitrite, high pH, and hypoxia on the aerobic metabolism of *C. auratus gibelio*. The results showed that the standard metabolic rate (SMR) was positively correlated with ammonia, nitrite, and pH, while the maximum metabolic rate (MMR) was negatively correlated with all four stressors. Thus, aerobic scope (AS) was reduced when *C. auratus gibelio* was exposed to high ammonia, high nitrite, high pH, and hypoxia. The peak of post-prandial O_2_ consumption was positively correlated with nitrite, pH, and the occurrence of the peak metabolic rate post-prandial was delayed in high ammonia, high nitrite, hypoxia, and high pH conditions. These findings indicated that, in experimental conditions, exposure to these environmental stressors can influence aerobic metabolism in *C. auratus gibelio*. With more energy required to maintain standard metabolic rates, less will be available for growth. While the *C. auratus gibelio* is one of the most hypoxia tolerance species, the reduction we observed in AS caused by stressors that commonly occur in ponds and in nature will likely affect growth in ponds and fitness in nature. These data have provided insight into the optimal, fitness-maximizing thresholds for these common stressors in this species of interest.

## 1. Introduction

When too high or too low, ammonia, nitrite, pH, and oxygen can become stressors for aquatic animals in traditional pond aquaculture and natural water bodies. Physiological activities of fish are susceptible to physical and chemical environmental stressors, which can be reflected by changes in metabolic activity [1,2,3,4].

Ammonia is excreted by fish as a nitrogenous waste product. In feed-based aquaculture, only 20% to 40% of the nitrogen in the protein of feed used in aquaculture ponds is recovered in harvest biomass. The other 60% to 80% remains in the water as uneaten feed, or is deposited as feces or excreted as ammonia nitrogen by aquatic animals [5]. Ammonia exists as unionized ammonia (NH_3_) and ionized ammonium (NH_4_^+^) in water. The toxicity of ammonia comes from the unionized form (NH_3_), which can diffuse across gill membranes due to its lipid solubility and lack of charge. While fish can excrete ammonia as NH_3_ across gill membranes into water [6], high environmental ammonia reduces the outward flux of ammonia through the gills. As a result, blood and tissue ammonia levels can increase and fish can experience both the chronic and acute effects of ammonia toxicity [7].

Ammonia produced by fish can be eliminated by bacteria that convert it to nitrite and nitrate. Nitrite is one of the most common toxic nitrogenous compounds in aquaculture systems, it can accumulate during intensive aquaculture due to the excessive use of proteinaceous feed, higher stocking densities, and/or imbalances between bacterial nitrification and denitrification. Elevated concentrations of nitrite can be toxic to aquatic animals, acting by mechanisms that have been investigated in various freshwater species, showing that elevated concentrations of nitrite in pond water can affect growth, molting, immune response [8], and ammonia excretion [9,10].

Dissolved oxygen (DO) is the primary limiting factor that dominates ectotherm physiology and behavior in aquatic ecosystems. DO regularly shows large fluctuations within the aquatic environment, especially in high density aquaculture. Hypoxia can result in reduced food consumption, slowed growth rates, reduced fecundity, or even death [11,12,13]. For these reasons, the impact of reduced environmental DO on the eco-physiology of fish must be studied and well understood.

Likewise, pH plays an important role in the maintenance of homeostasis in aquatic animals [14]. High pH water can cause immediate, dramatic inhibition of ammonia excretion and a subsequent increase in plasma ammonia [4,14], which is potentially lethal. However, the physiological and behavioral responses of *C. auratus gibelio* to alkaline conditions have yet to be studied.

Measurements of the mass specific rate of O_2_ consumption (MO_2_) have become common in research on fish biology and climate change, largely due to renewed interest and new developments that have related an aerobic scope (the range between minimum and maximum MO_2_, respectively) to whole-animal performance and fitness [15]. MO_2_ is influenced by various factors such as body mass, temperature, food intake, physiological state, activity level, and anabolism. The three fundamental metabolic variables are standard metabolic rate (SMR) [16], maximum metabolic rate (MMR), and aerobic metabolic scope (AS) [17]. SMR and MMR are usually calculated using measurements of oxygen consumption rate. The SMR is the minimum MO_2_, which allows for no activity, digestion, growth, or production of sexual products, as it represents the basic cost of being alive and is of major functional importance. If the MO_2_ is below the SMR, physiological function has been impaired in some way, and most species cannot survive for long in this state. The MMR, on the other end of the range, indicates the swimming speed and predation capability. Aerobic scope (AS), defined as the difference between the SMR and MMR, represents the oxygen usage capacity, which in turn indicates the total amount of aerobic energy available to the animal for processes including digestion, locomotion, growth, and reproduction.

*C. auratus gibelio*, is an omnivore that is popular because of its flavor, and is commonly used as a benthic species in Chinese pond polyculture [18]. It is a hypoxia-tolerant species but is relative sensitive to high nitrite and pH levels. To provide basic data for the physiology and metabolism of *C. auratus gibelio*, its aerobic metabolism in response to high ammonia, nitrite, pH, and hypoxia were evaluated during acute exposure.

## 2. Materials and Methods

*C. auratus gibelio* were acclimatized for 2 weeks in 300 L tanks at 26.0 ± 1 °C before experiments. During this period, the fish were fed daily using commercial pellets. Ammonia, nitrate, and pH levels were monitored and 1/3 of the water was changed every day. Total ammonia was 0.13 ± 0.02 mg L^−1^, nitrate was 0.17 ± 0.02 mg L^−1^, and pH was 8.2 ± 0.1. Body mass and length of the fish (means ± SE) were 15.3 ± 2.3 g and 9.0 ± 0.6 cm, respectively, and were not significantly different between treatment groups. All fish were collected under permits issued by local and national authorities, and experimental procedures were in accordance with national animal care regulations.

A custom-made respirometer was used for MO_2_ measurement. Prior to the experiment, fish underwent a fasting period of 48 h, enough to ensure that they were in a post-absorptive state. In the intermittent flow through respirometry, water was continuously circulated through each respirometer using an in-line submersible pump within a recirculation loop. Each chamber (2 L) was equipped with a recirculation pump that only turned on when the inflow water was turned off for a measurement to ensure sufficient water mixing and minimize intermittent disturbance to the fish.

The study included numerous independent treatments: (1) In the control group, fish were exposed to fresh holding water; (2) experimentally manipulated fish were exposed to high-ammonia water (0.5, 1.0, and 2.0 mg L^−1^), high-nitrite water (0.5, 1.0, and 1.5 mg L^−1^), hypoxic water (4.0 and 1.5 mg L^−1^, and 8.0 mg L^−1^ as a control), and high pH water (9.1, 9.5, and 9.9). Ammonia was adjusted by adding ammonium chloride, nitrite was adjusted by adding sodium nitrite, DO was controlled by mixing nitrogen and oxygen, and alkaline (high pH) water was prepared by adding sodium carbonate and sodium bicarbonate (total alkalinity was 30.0 m mol L^−1^).

### 2.1. Measurements of Background Respiration

Background respiration (to account for microbial respiration) was estimated by measuring the oxygen consumption rate in the respirometer without fish. Background respiration was measured before and after the experiment. Background oxygen consumption rates were used to correct fish MO_2_ values.

### 2.2. Measurement of Standard Metabolic Rate (SMR)

The duration of each treatment was 96 h, SMR was measured every 24 h. After an acclimation period (24 h), and oxygen consumption was measured every hour throughout a 96 h experimental period in each chamber (fish was put in the chamber singular). Dissolved oxygen (DO) was monitored every second using an oxygen meter (YSI 6600, measuring precision is 0.01 mg L^−1^), and the oxygen probe was calibrated before each experiment. The experiment was conducted in a dark environment. The fish appeared to be in excellent condition and remained quiet while measurements were made in the dark. Each group had three parallel replicates, with three fish in each.

### 2.3. Measurement of Maximum Metabolic Rate (MMR)

The duration of each treatment was 96 h, MMR was measured every 24 h. MMR was measured using an exhaustive chase protocol, where the experimenter manually chased the fish to exhaustion (5 min). All individuals were visibly exhausted by the end of the 5 min exercise period as highlighted by a lack of response to an experimenter tapping the caudal fin. This was followed by a period of exposure to air (approximately 1 min), with the goal of completely exhausting the fish. Measurements of MO_2_ began immediately after the fish was moved to a respirometer that was quickly sealed (within 20 s). The duration of the measurement was 9 min. Once MMR had been determined for each fish, the respirometers were set to the automated flush cycles outlined above and MO_2_ was measured for at least 6 h while the fish recovered. Each group had three parallel replicates, with three fish in each.

### 2.4. Measurement of Post-Prandial MO_2_

Post-prandial MO_2_ was measured after 96 h exposure of each treatments. Fish were fed 1% of their body weight daily, using the same feed as during holding. Any regurgitated pellets were counted and, in each case, were determined to be negligible relative to the size of the meal. Thirty minutes after feeding, the fish was replaced into their chamber and monitored for a further 24 h. MO_2_ was measured every hour for the duration of the 96 h experimental period. Each group had three parallel replicates, with three fish in each. Peak post-prandial MO_2_ was determined for each fish that had continuous measurements as the highest hourly block value. Time-to-peak post-prandial MO_2_ was the number of hours post-prandial it took to reach the peak value.

### 2.5. Statistical Analysis

The lowest 10% MO_2_ values were used to calculate SMR. The SMR (mg/(kg h)) of each fish was calculated according to the following equation:

*SMR* = (*DO_k_* − *DO_k_*_+1_)*V*/(*t* × *m*)

where *DO_k_* and *DO_k_*_+1_ are the oxygen concentration (mg L^−1^) at point k and point *k* + 1, respectively; V (L) is the total volume of the respirometer (2 L) minus the volume of the fish; *t* (h) is the interval (5/60 h) between points *k* and *k* + 1; m (kg) is the body mass of the fish.

MMR was measured after an exhaustive chase protocol, MO_2_ was measured for 9 min, and MMR was taken as the steepest three min slope during this time. Subtracting SMR from MMR provided the AS value.

Within each acclimation group, a one-way ANOVA was performed. Results were considered statistically significant when *P* < 0.05, and all results are presented as means ± SE. Statistically significant differences in SMR, MMR, AS, and peak post-prandial MO_2_, starting from the post-transfer 0 h, among treatments at each time point were revealed using one-way ANOVAs, followed by multiple sample comparisons using the Holm–Sidak method. Nonlinear regression was used to analyze the correlation between concentration of treatments and SMR, MMR, or AS. Differences were considered significantly different when *P* < 0.05. SAS9.3 (SAS Institute, Cary, NC, USA) was used for all statistical analyses. Line charts were created using SigmaPlot 11.0 (Systat Software, San Jose, CA, USA).

## 3. Results

### 3.1. Effects of High Ammonia on Aerobic Metabolism

**Figure 1:**
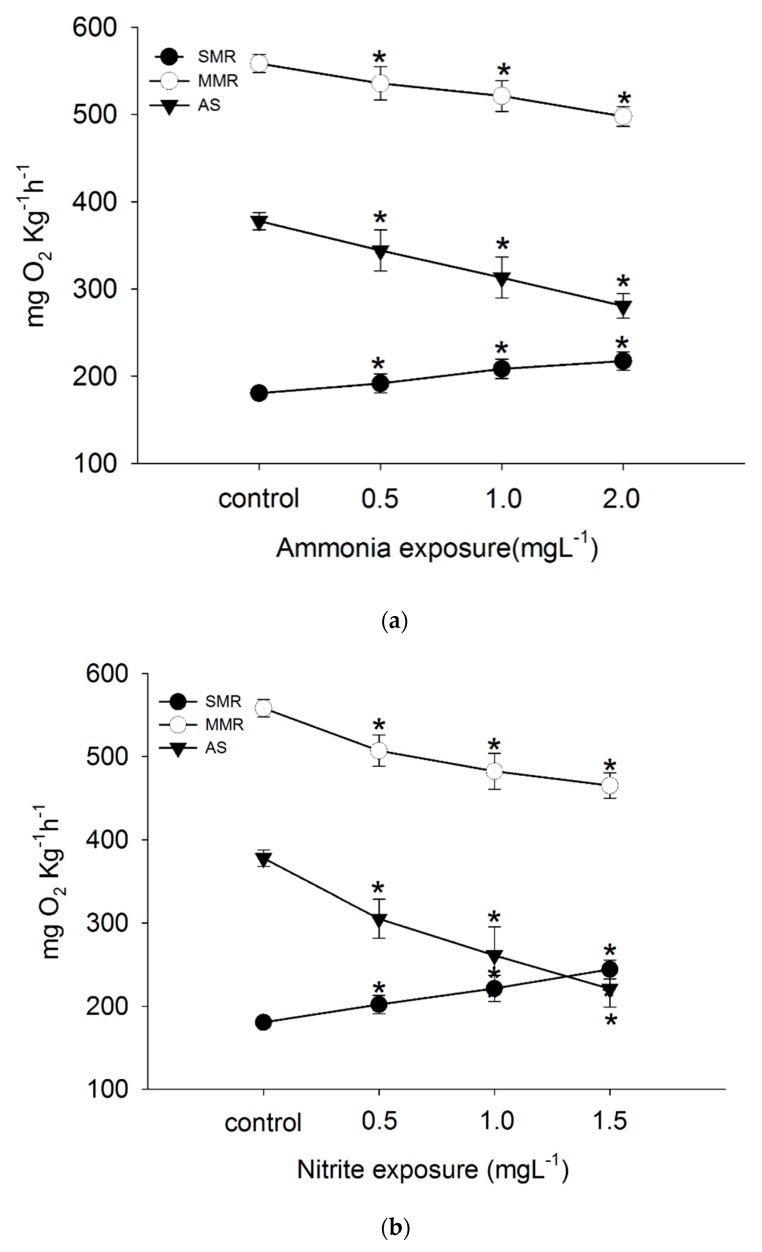

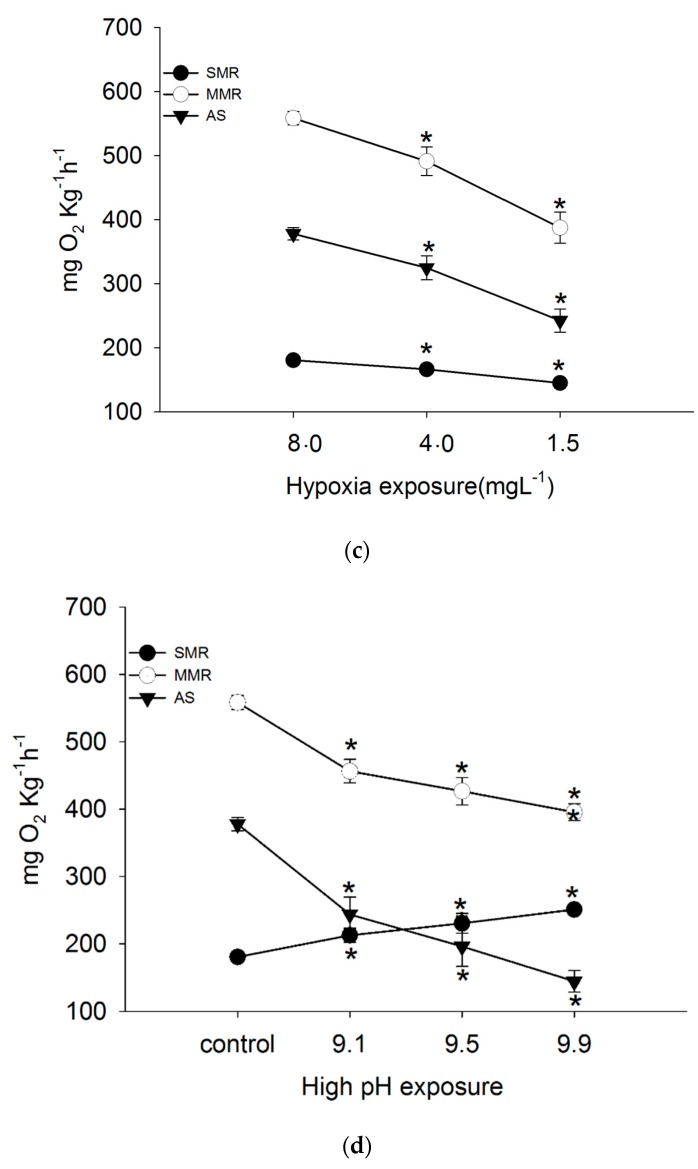
Standard metabolic rate (SMR), maximum metabolic rate (MMR), and aerobic scope (AS) of *Carassius auratus gibelio* exposed to high ammonia (control, 0.5 mg L^−1^, 1.0 mg L^−1^, 2.0 mg L^−1^) (**a**), high nitrite (control, 0.5 mg L^−1^, 1.0 mg L^−1^, 1.5 mg L^−1^) (**b**), hypoxia (control, 4.0 mg L^−1^, 1.5 mg L^−1^) (**c**), and high pH (control, 9.1, 9.5, 9.9) (**d**). Values are means ± SE. Statistically significant difference among treatments at each time point (“*”shows the significant different from control) were revealed by a one-way ANOVA test, followed by multiple comparisons with the Holm–Sidak method (*P* ≤ 0.05).

**Figure 2:**
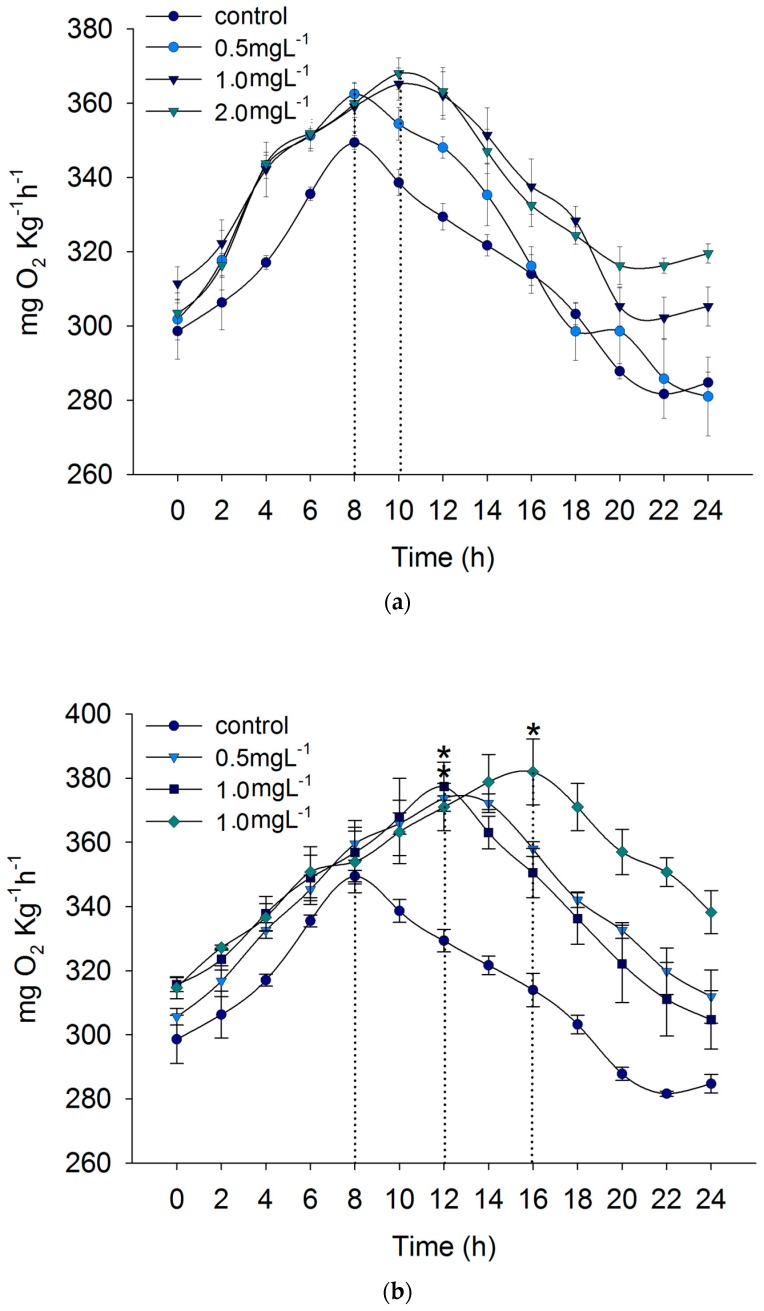

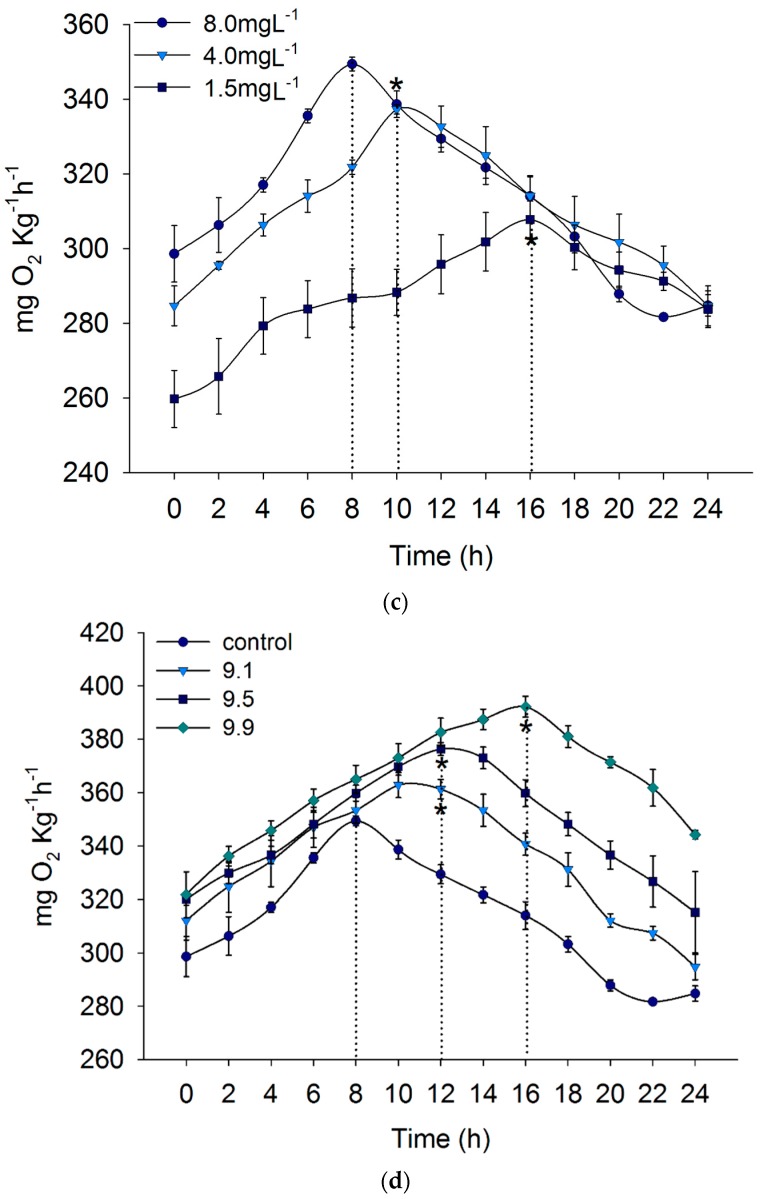
Post-prandial MO_2_ of *Carassius auratus gibelio* exposed to high ammonia (control, 0.5 mg L^−1^, 1.0 mg L^−1^, 2.0 mg L^−1^) (**a**), high nitrite (control, 0.5 mg L^−1^, 1.0 mg L^−1^, 1.5 mg L^−1^) (**b**), hypoxia (control, 4.0 mg L^−1^, 1.5 mg L^−1^) (**c**), and high pH (control, 9.1, 9.5, 9.9) (**d**).Values are means ± SE. Statistically significant difference among treatments at each time point (“*”shows the significant different from control) were revealed by a one-way ANOVA tests, followed by multiple comparisons with the Holm–Sidak method (*P* ≤ 0.05).

SMR of *C. auratus gibelio* increased significantly with increasing ambient ammonia (*P* < 0.05). The relationship between SMR and ambient ammonia was quadratic (Figure 1a). In the 2.0 mg L^−1^ ammonia environment, SMR increased by 20.4% compared to the control. MMR decreased significantly with increasing ambient ammonia (*P* < 0.05). The relationship between MMR and ambient ammonia was exponential. In the 2.0 mg L^−1^ ammonia environment, the MMR decreased by 10.8% compared to the control (Figure 1a). AS decreased significantly with increasing ambient ammonia (*P* < 0.05). The relationship between AS and ambient ammonia was quadratic. In the 2.0 mg L^−1^ ammonia environment, AS decreased by 25.0% compared to the control (Figure 1a). The time to peak post-prandial MO_2_ was delayed with increasing ambient ammonia (Figure 2a).

### 3.2. Effects of High Nitrite on Aerobic Metabolism

SMR of *C. auratus gibelio* increased significantly with increasing ambient nitrite (*P* < 0.05). The relationship between SMR and ambient nitrite was quadratic. In the 1.5 mg L^−1^ nitrite environment, SMR increased by 35.3% compared to the control (Figure 1b). The MMR decreased significantly with increasing ambient nitrite (*P* < 0.05). The relationship between MMR and ambient nitrite was quadratic. In the 1.5 mg L^−1^ nitrite environment, the MMR decreased by 16.7% compared to the control (Figure 1b). AS decreased significantly with increasing ambient nitrite (*P* < 0.05). The relationship between AS and ambient nitrite was quadratic. In the 1.5 mg L^−1^ nitrite environment, AS decreased by 41.5% compared to the control (Figure 1b). Peak post-prandial MO_2_ increased with increasing ambient nitrite (*P* < 0.05), and time to peak post-prandial MO_2_ was delayed with increasing ambient nitrite (Figure 2b).

### 3.3. Effects of Hypoxia on Aerobic Metabolism

SMR of *C. auratus gibelio* increased significantly with increasing ambient dissolved oxygen (*P* < 0.05). The relationship between SMR and ambient dissolved oxygen was logarithmic. In the 1.5 mg L^−1^ dissolved oxygen environment (hypoxic), SMR decreased by 19.7% compared to the control (Figure 1c). The MMR increased significantly with increasing ambient dissolved oxygen (*P* < 0.05). The relationship between MMR and ambient dissolved oxygen was logarithmic. In the 1.5 mg L^−1^ hypoxia treatment, the MMR decreased by 30.6% compared to the control (Figure 1c). AS decreased significantly with decreasing ambient dissolved oxygen (*P* < 0.05). The relationship between AS and ambient dissolved oxygen was logarithmic. In the 1.5 mg L^−1^ dissolved oxygen, AS decreased by 35.9% compared to the control (Figure 1c). Peak post-prandial MO_2_ increased with decreasing dissolved oxygen (*P* < 0.05), showing an inverse relationship. Time to peak post-prandial MO_2_ was delayed with decreasing dissolved oxygen (Figure 2c).

### 3.4. Effects of High pH on Aerobic Metabolism

SMR of *C. auratus gibelio* increased significantly with increasing ambient pH (*P* < 0.05). The relationship between SMR and ambient pH was exponential. In the 9.9 pH environment, SMR increased by 40.0% compared to the control (Figure 1d). The MMR decreased significantly with increasing ambient pH (*P* < 0.05). The relationship between MMR and ambient pH was logarithmic. In the 9.9 pH environment, the MMR decreased by 23.4% compared to the control (Figure 1d). AS decreased significantly with increasing ambient pH (*P* < 0.05). The relationship between AS and ambient pH was quadratic. In the 9.9 pH environment, AS decreased by 61.8% compared to the control (Figure 1d). The peak post-prandial MO_2_ increased with increasing pH (*P* < 0.05) and had a quadratic relationship with pH. The time to peak post-prandial MO_2_ was delayed with increasing pH (Figure 2d).

## 4. Discussion

While being one of the most hypoxia tolerance species, *C. auratus gibelio*’s AS was reduced by key stressors such as high ammonia, high nitrite, hypoxia, and high pH, that occur unpredictably in ponds and nature. A reduction in AS can lead to a reduction in growth in aquaculture ponds or reduced fitness in nature. The results described herein have provided novel insights into the optimal, fitness-maximizing thresholds of stressors for this economically valuable species.

### 4.1. Effects of High Ammonia on Aerobic Metabolism

High ammonia impaired the aerobic metabolism of *Carassius auratus gibelio.* AS is used for locomotion and growth and was reduced as a function of the increase in SMR and decrease in MMR. A previous study suggested that the mechanism of ammonia poisoning in fish might be that ammonia interferers with amino acid transport, causing the swelling of astrocytes in the brain, disrupting the metabolism of amino acid neurotransmitters [19]. Fish attempt to excrete ammonia by energy-dependent transporters, such as V-type H^+^-ATPase [20] and Na^+^/K^+^-ATPase [21]. So, if the *C. auratus gibelio* needs more energy to excrete ammonia to defend against hyperammonemia, its supply of energy available to grow will be reduced. In addition, MMR and AS both decreased after exposure to ammonia, which may result in difficulty getting food and being more easily caught by predators. Although peak post-prandial MO_2_ was not different among treatments, delayed time-to-peak post-prandial MO_2_ indicated that *C. auratus gibelio* required more time for food ingestion. Due to the unforgiving nature of the wild, once this fish cannot meet its basic requirements, it may more easily become diseased.

### 4.2. Effects of High Nitrite on Aerobic Metabolism

High nitrite impaired the aerobic metabolism of *C. auratus gibelio*, which may translate to reduced locomotion and growth because of the increased SMR and decreased MMR, meanwhile, peak post-prandial MO_2_ and time-to-peak post-prandial MO_2_ both increased. The relative increase in peak post-prandial MO_2_ for *C. auratus gibelio* exposed to high nitrite reached a maximum of 2.1 times higher than SMR. Post-prandial MO_2_ represents the metabolic expenditures resulting from the nutritive process but may also include components relating to the energy requirement for ingestion, which varies among fish species and has been shown to be dependent upon several factors [22,23,24]. The increase of peak post-prandial MO_2_ and time-to-peak post-prandial MO_2_ were consistent with low feeding rate and food conversion efficiency [25]. When metabolism increases while the AS is narrower, the fish becomes more sensitive to hypoxia during digestion and may reduce ingestion. One physiological response to nitrite is an increase in methemoglobin. The hemoglobin becomes oxidized and unable to bind and carry molecules of oxygen [26]. In this case, the increased SMR in *C. auratus gibelio* was a result of either the induction of mitochondria dysfunction, or the increased energy demand by repair mechanisms and toxicant elimination [27]. The decreased MMR might be one side of a “trade-off” between the metabolic costs of chemical detoxification and fish activity.

### 4.3. Effects of Hypoxia on Aerobic Metabolism

Hypoxia impaired the aerobic metabolism of *C. auratus gibelio*, AS decreased because of the decrease in MMR, while peak post-prandial MO_2_ decreased and time-to-peak post-prandial MO_2_ increased in the hypoxic environment. SMR, MMR, and AS of *C. auratus gibelio* decreased when exposed to hypoxic conditions. We suggest, therefore, that *C. auratus gibelio* responses to environmental hypoxia were based mainly on suppressed metabolic rate. Fish of the genus Carassius evolved a specialized metabolic system that allows them to survive prolonged periods in hypoxia, even without oxygen by producing ethanol as their metabolic end-product [28]. Increased time-to-peak post-prandial MO_2_ indicate that although *C. auratus gibelio* survives in hypoxia by low metabolic rate, they need more time for digestion.

### 4.4. Effects of High pH on Aerobic Metabolism

High pH impaired the aerobic metabolism of *C. auratus gibelio*, AS decreased because of the increase in SMR and decrease in MMR, while peak post-prandial MO_2_ and time-to-peak post-prandial MO_2_ both increased. SMR decreased when exposed to high pH, this was likely because high pH water affected ion equilibriums and ammonia excretion [29,30]. If excretion of ammonia was limited, the fish would need more energy to force excretion or utilize other energetically expensive ways to excrete ammonia, which would cut into the energy available for growth. Meanwhile, high pH and alkalinity could cause an acid base imbalance, such as respiratory alkalosis [4], thus demanding greater energy to cope with the disturbance. The relative increase in peak post-prandial MO_2_ for *C. auratus gibelio* exposed to high pH reached a maximum of 2.2 times higher than SMR, along with extremely narrow AS, which may result in difficulty getting food and a low ingestion rate. This is consistent with our findings about the growth performance of fish in alkaline water [31]. These findings give us some hint that extra oxygen should be provided when farming the fish in high pH water, especially during the feeding and ingestion period.
